# Attention Allocation to Financial Information: The Role of Color and Impulsivity Personality Trait

**DOI:** 10.3389/fnins.2019.00818

**Published:** 2019-08-06

**Authors:** Maria G. Ceravolo, Rocco Cerroni, Vincenzo Farina, Lucrezia Fattobene, Lucia Leonelli, Nicola B. Mercuri, GianMario Raggetti

**Affiliations:** ^1^Department of Experimental and Clinical Medicine, School of Medicine, Marche Polytechnic University, Ancona, Italy; ^2^Neuroeconomics Laboratory, Centre for Health Care Management, School of Medicine, Marche Polytechnic University, Ancona, Italy; ^3^Neurological Unit, Department of Systems Medicine, University of Rome “Tor Vergata”, Rome, Italy; ^4^Department of Management and Law, School of Economics, University of Rome “Tor Vergata”, Rome, Italy; ^5^IRCCS Santa Lucia Foundation, European Centre for Brain Research, Rome, Italy

**Keywords:** neurofinance, financial information processing, eye tracking, visual attention, eye movements, impulsivity, color

## Abstract

**Background:**

In order to raise the level of investor’s protection, the European Commission has recently introduced the key investor information document (KIID), a standard, plainly worded, and consumer-friendly document which should provide individuals with essential information for their investment decisions. KIID layout has been delineated relying on results from focus groups, surveys and telephone interviews, ignoring the reliable, and unbiased insights offered by neuroscientific approaches.

**Aim:**

The current study aims to elucidate the patterns of eye movements in the early phases of information acquisition during the reading of financial disclosure documents, disentangling the independent role of color and impulsivity at modulating attention distribution toward the different sources of financial information.

**Materials and Methods:**

Oculomotor behavior was monitored in eighty-one healthy adults through the eye tracking technology (SMI REDn Scientific 60 Hz). An ecological protocol exploiting KIIDs was developed to control for several individual variables, through delivering standard visual stimuli based on official financial documents. Participants performed a passive exploration task (with the only specific instruction of visually exploring the document), followed by an active task where they were asked to rate the financial attractiveness of the products, as low, medium or high. The Barratt impulsiveness scale (BIS-11) was administered to each participant, to score such personality trait.

**Results:**

Attention distribution over the different information sources, included in the KIIDs, has been quantified and found to be independently modulated by both color and impulsivity, with a greater role of the former over the latter. The addition of either red or blue information to some KIID’s sections increases attention allocation toward the whole document, compared to the usual black and white version. BIS-11 total scores were inversely related to the first and average fixation duration in the neutral, though not in the colored condition.

**Discussion:**

Bottom-up, stimulus-related mechanisms of attention allocation influence information acquisition during the reading of financial prospectuses to the point that the increased attention induced by color compensates for individual impulsivity. Such evidence should be considered by regulators when devising the disclosure documents in order to increase investors’ protection.

## Introduction

A key mechanism for evolutionary success is the ability to gather relevant information, discarding irrelevant one. The neural function that enables this crucial process is called *selective attention*. Among the five senses, a major role is played by the visual sensory system: a large part of the afferents is comprised by the optic nerves, supporting the leading role of visual attention in guiding human behavior. It is through saccadic eye movements that bring the retinal region of highest acuity, the fovea, onto the stimuli, that we attend to and look at objects in the visual field. What we pay attention to in the visual environment is the result of the dynamic interaction among *bottom-up* processing, that proceeds in a single direction from sensory input to motor output, and *top-down* processing, that is related to the flow of information which conveys knowledge derived from previous experience, expectations and goals, and also factors outside these categorization as unexpectedness and novelty, which reflect the interaction between sensory and cognitive influences (Corbetta and Shulman, 2002). Psychological evidence points out that attention and eye movements are functionally related but it has not been clarified yet to what extent these processes share the same underlying computations and neural systems (Corbetta et al., 1998). According to Corbetta and Shulman (2002), visual attention mechanisms are controlled by two segregated neural systems: one includes parts of the intraparietal cortex and superior frontal cortex and is involved in preparing and applying goal-directed (top-down) selection for stimuli and responses; the other (mainly lateralized to the right hemisphere) includes the temporoparietal cortex and inferior frontal cortex, and is specialized for the detection of behaviorally relevant stimuli, particularly when they are salient or unexpected. This second network works as a ‘circuit breaker’ for the dorsal system, directing attention to salient events. Both attentional systems interact during normal vision, and both are disrupted in unilateral spatial neglect.

Recent investigations suggest that the visual selection and oculomotor programming might be integrated during visually guided behavior (Rizzolatti et al., 1987; Hoffman and Subramaniam, 1995; Kowler et al., 1995; Awh et al., 2006). Even if evidence disproves any causal relationship between eye movements and preference formation, eye movements are still considered factors of down-stream effects on decision making (Orquin and Mueller Loose, 2013).

Since financial information available to investors is abundant, it is impossible to attend entirely to it, but the attendance toward some sources of information rather than others clearly impacts on financial market, driving the need to explore investors’ attention allocation process. According to Frydman and Camerer (2016), only a few studies measure financial attention directly, i.e., trough eye-tracking, while most of them use proxies as Google searches of individual stock ticker symbols. As a matter of fact, very few studies have applied the eye tracking technology to shed light on the neural mechanisms that underlie investor’s behavior (Shavit et al., 2010; Hüsser and Wirth, 2014; Duclos, 2015; Rubaltelli et al., 2016), and none of them has focused on attentional mechanisms during financial information processing considering also the influence of the color of stimuli and of the impulsivity personality trait.

Color is largely used in the financial sectors (e.g., in websites, television, newspapers, financial reports, trading platform, and security market displays), where its influence on attention and decision making has been frequently investigated (Farley and Grant, 1976; Clifford et al., 2010). Red color priming emphasizes value losses of the underlying asset by leading to assign higher probabilities to events involving the loss domain (Kliger and Gilad, 2012). The use of color impacts on trading decisions (Bazley et al., 2019), suggesting that the representation of financial information, rather the information *per se*, impacts on investors’ decision making. Also the presentational format of financial disclosure documents modulates subjects’ attention allocation (Ceravolo et al., 2018), so that, when the section concerning the costs is placed in the top left quarter, attention toward the whole document increases, with a significant effect on the attractiveness evaluations about products.

Concerning the role of impulsivity personality trait in modulating the attentional networks, back in 1965, Shapiro suggested that a short-circuiting of attention is related to impulsive behavior while Dickman (1993) argued that individual differences in impulsivity could be the result of differences in the attention allocation mechanisms. Even if impulsivity is generally associated to less forethought action, impulsive subjects tend to respond more slowly in some experimental tasks, and compared with less impulsive individuals. Specifically, less impulsive individuals perform better on tasks which require fixation of attention while highly impulsive ones in those that require attention shifts (Dickman, 1985). Expósito and Andrés-Pueyo (1997) reported that more impulsive subjects showed significantly greater response latencies, in a choice task, than less impulsive individuals. According to Redgrave et al. (1999), a greater ability to shift spatial attention, i.e., the allocation of attentional resources to new and unexpected stimuli (which can be aversive or rewarding) are driven by a short duration burst of firing in mesencephalic dopaminergic neurons. Reward stimuli have been particularly investigated with respect to impulsive subjects’ ability to shift from one stimulus to another and it has been found that impulsive individuals focus more on reward targets and less on neutral spatial locations, than less impulsive individuals (Ávila and Parcet, 2002). Analyzing the relationship between impulsivity and covert visual orienting of attention guided by peripheral or central cues, Poy et al. (2004) found out that impulsivity facilitates conscious shifting of attention when covert orienting is guided by expectation, so that impulsive individuals are faster in shifting visual spatial attention when the target appears in an unexpected location. A possible explanation relies in the role played by dopamine that predisposes to switch attentional resources toward unexpected or salient stimuli (Avila et al., 2003). To the best of our knowledge, there is no data so far concerning the possible influence of colored financial information on attention allocation measured through the analysis of oculomotor behavior and taking into account individual differences in impulsivity. It would be extremely interesting to demonstrate that a relationship exists between colored stimuli, impulsivity and visual attention also in the financial domain, where computing competences are required and emotions are expected to play a minor role.

In the present study, we aim to contribute to the knowledge about stimulus-driven attentional mechanisms by using eye tracking to analyze the patterns of eye movements in the phase of information acquisition during the reading of financial disclosure documents. We sought to investigate whether the use of color modulates attention distribution toward the different sections of financial information (both colored and not colored), while controlling for individual impulsivity trait.

## Materials and Methods

### Participants

A total of 90 participants with normal or corrected to normal vision and no report of eye or neurological diseases volunteered for the experiments. They were all Italian undergraduate students, with basic data analysis skill (as documented by successful completion of a Statistics course), and who declared no previous exposure to financial documents and no gambling attitude. Data from 9 participants were discarded because eye tracking score for calibration and validation were below the acceptance threshold. The final sample consisted of 81 participants (age: 24 ± 2 years). Out of 81, 40 (age: 23 ± 2; females = 15) were random assigned to read black and white documents and 41 (age: 24 ± 2; females = 26) were assigned to read colored documents.

This study was carried out in accordance with the recommendations of the Institutional Review Board of the University of Rome Tor Vergata. All subjects gave written informed consent in accordance with the Declaration of Helsinki, after receiving an explanation of the procedure and aims of the study, which was approved by the quoted ethics committee.

### Visual Stimuli and Task

The experimental protocol was created and implemented through SensoMotoric Instruments (SMI GmbH) *Experiment Center*. It was composed of 18 visual stimuli, representing as many key investor information documents (KIIDs), displayed on a laptop screen for maximum 60 s each. Participants were allowed to move forward to the next stimulus as soon as they felt to have acquired all the necessary information, by simply pressing the space bar. This choice was made to prevent subjects’ fatigue, or boredom, and a subsequent attention decrease. To increase subjects’ involvement and motivation we asked them to rate the financial attractiveness of each product.

**FIGURE 1:**
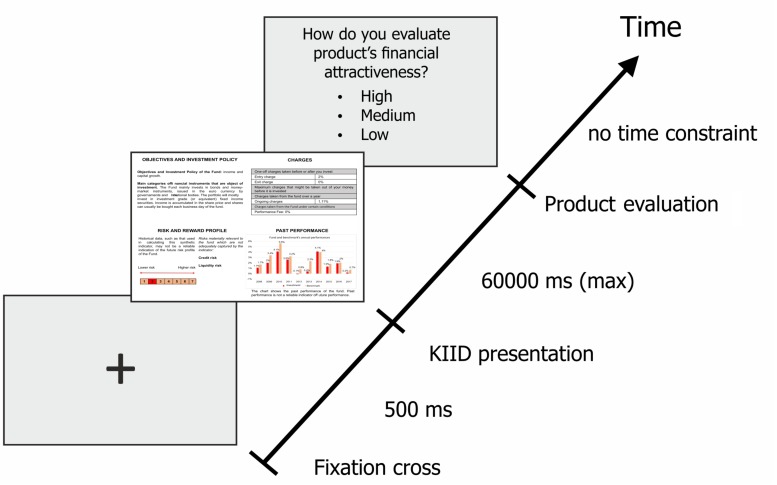
Eye-tracking protocol. The sequence starts with a blank screen with a cross in the middle, followed by the presentation of a KIID that the subject has to scan in maximum 60 s before proceeding to the next slide, where he is asked to provide his judgment of financial attractiveness. The whole sequence is repeated 18 times.

Therefore, the request of rating the financial attractiveness as high, medium, or low, was displayed after each product presentation. The response was delivered by using the laptop touchpad and pressing the key on the corresponding value; the system immediately presented the next stimulus. Once the answer was entered, participants were unable to change their responses. There was no time constraint in delivering the answer. Figure 1 illustrates the experimental procedure.

The visual stimulus used in the experiment was adapted from the official financial disclosure document introduced by the recent UCITS IV Directive: the KIID. The layout and the content of this compulsory, pre-contractual prospectus have been standardized in order to allow comparability of financial products across European countries and to provide retail investors with sufficient information to assume informed investment decisions. The original predetermined format of the KIID, pursuant to guidelines issued by European Securities and Markets Authority (ESMA), consists of the following features: two A4 pages in length, a minimum font size of 8, and five key sections to be displayed in the running order of the ESMA template, as follows:

(a) *Objectives and investment policy* (*Objectives*, hereinafter): a narrative description, in plain language, of the main current, and expected investment policy and of the main categories of financial instruments where the fund manager intends to invest.
(b) *Risk and reward profile (RRP)*: a section that includes a synthetic indicator risk and reward graphic (SRRI), i.e., a numerical scale ranging from 1 to 7 (where 1 indicates a low and 7 a high risk and reward potential) based on the volatility of a UCITS’ past performance. The section also presents a short narrative description of the composition of the SRRI and of the risks that it does not capture.
(c) *Charges for Buyer:* it presents the entry, ongoing and exit charges, in a tabular format.
(d) *Past performance:* graphical representation of the UCITS’ 10 years past performance, compared to a benchmark. This section also includes a *Disclaimer* informing that past performances are not predictive of future ones.
(e) *Practical information:* in order to balance the experimental requirement of standardized visual stimuli and the need to reproduce financial documents close to real scenarios for investment decisions, we exploited the information from official KIIDs while taking several precautions in building the final stimulus. Even if branding is permitted by the Directive, as long as it does not obscure text or distract investors from the essential contents, we deleted the name of the fund and related financial institution in order to rule out any influence of the image and the associated reputation. Thus, the practical information section was not presented. We standardized the number of text lines in each section and the font size (that was 11), in order to allow comparability of oculomotor patterns across the different stimuli. In the performance section, we always displayed the standardized time span of the last 10 years from 2018, in order to reduce inter-individual variability at inferring the ability of the funds’ management to be profitable and survive. With respect to the SRRI, we avoided to display KIIDs exhibiting extreme values (like 1 and 7), to avoid that an anchoring effect could strongly affect attentional allocation and the individual rating of product financial attractiveness.

Subjects were random assigned to be exposed to either neutral vs. colored stimuli. Apart from the color, all the remaining features of the visual stimuli (including the sequence) were the same for all subjects.

In the neutral condition, the standardized KIIDs were drafted in black and white. In the colored condition, visual stimuli were built using either blue, or red. The Directive neither precludes the use of colors to present these financial disclosure documents, nor it recommends any specific color to be used when drafting them. After an extensive screening of the available KIIDs, we found that a large part of documents is drafted either in blue or red. Therefore, red and blue were selected, in alternative to the plain black and white version, to construct visual stimuli applied in this experiment. Moreover, in order to mimic the real-life scenario, where color just applies to elements as the SRRI and the graph of the past performance, we only provided stimuli where the colored sections were the quoted ones.

### Apparatus

The SMI REDn Scientific System, which uses infrared light sources and cameras that are integrated into a 15.6-inch monitor (1280 × 1024 pixels), was used. By means of corneal reflection techniques, the SMI system records the *X* and *Y* coordinates of each participant’s eye position at 60 Hz rate (i.e., 3600 data collections/min). Testing was performed in a research laboratory. Participants’ distance from the eye tracking device was between 60 and 80 cm. The system compensates for head movements within a 50 cm × 30 cm (at 65 cm distance), allowing the participants to look at the screen in a naturalistic manner. The tracker has a reported gaze position accuracy of 0.4° and a spatial resolution of 0.05°. The task was preceded by a 5-point calibration procedure, which was repeated until calibration was sufficient for each of the data points. The proper calibration of the eye tracker was re-validated and, if necessary, re-calibrated in the middle of the experiment. Prior to each trial, a light gray slide with a fixation-cross appeared for 2 s to reorient attention and ensure that all scanning patterns began from the center of the screen.

### Eye Tracking Parameters

The following gaze metrics were considered:

- *Entry Time:* expresses the average interval (ms) from the presentation of the KIID document (start of the trial) to the first gaze fixation on each area of interest (AOI).
- *Dwell Time (DT)*: is the total duration (ms) of all fixations and saccades within an AOI for all subjects, divided by number of subjects.
- *Dwell Time Percentage (DT%)*: equals the DT multiplied by 100 and divided by the difference between current time and start time of the trial.
- *Normalized Dwell* (*ms/coverage*) (*NormD*)*:* is the ratio between the DT and the AOI coverage, where coverage is the AOI size (measured in pixel) in comparison to stimulus size, thus representing a percentage of the number of pixel (px). It represents a more reliable measure to understand attention distribution patterns, since it adjusts the duration that a subject spent to process an item relative to its surface in the display.
- *First Fixation Duration (FFD):* duration (ms) of the first fixation to hit the AOI.
- *Average Fixation Duration (AFD):* is the total duration (ms) of all fixations divided by the number of fixations inside the AOI. A longer fixation duration is often associated with a deeper and more effortful cognitive processing.
- *Revisits:* is the number of time subjects visit an AOI.

### Personality Inventory – BIS-11

The Barratt impulsiveness scale (BIS-11) (Patton et al., 1995) questionnaire was administered to all study participants. The BIS-11 is a questionnaire designed to investigate the personality/behavioral construct of impulsiveness. It is the most widely cited instrument for the assessment of impulsiveness and has been used to advance our understanding of this construct and its relationship to other clinical phenomena for 50 years.

The current version of BIS-11 is composed of 30 items describing common impulsive or non-impulsive (for reverse scored items) behaviors and preferences. Items are scored on a 4-point scale, where 1 point is associated to “Rarely/Never”, 2 points to “Occasionally”, 3 points to “Often,” and 4 points to “Almost Always/Always”.

The perspective of Doctor Barratt and International Society for Research on Impulsivity is that impulsivity is a multi-faceted construct and this multi-dimensionality is reflected in the BIS-11 factor structure. Therefore, in the BIS-11, not only the total score, but at least the second-order factors should be reported to help considering their individual contribution to personality assessment (Patton et al., 1995).

Impulsivity is therefore considered an expression of heterogeneous phenomena, so much that Barratt proposes a subtyping of impulsive behavior, assuming the existence of three subtypes of impulsivity: motor impulsivity, defined as the innate tendency to act before thinking (motor activation); cognitive/attentional impulsivity, such as the tendency to make rapid decisions, and the lack of concentrations with respect to the task (inattention); non-planning impulsivity, which would be defined as a behavioral modality characterized by a scarce assessment of the consequences (lack of planning).

### Statistical Procedure

Eye-position data were analyzed with a standard area-of-interest (AOI) approach. Specifically, rectangular AOIs were defined over the described four main sections of the stimulus (*Objectives, RRP, Charges*, and *Past performance*) and over four minor sections, i.e., the *SRRI*, the *Disclaimer* and two sub-sections of the past performance corresponding to the *First years* and the *Last years* of the whole time span. Eye tracking data were pre-processed using the SMI software named *BeGaze.* Different aspects of eye movements were assessed. We included 6 dependent variables in our eye-tracking analyses: dwell time, dwell time percentage, normalized dwell, average fixation durations, first fixation duration, and revisits. For each of our dependent variables, we ran an ANOVA with the experimental condition (neutral vs. colored) as grouping variables. In order to outline the independent role of BIS-11 score at modulating attention distribution, simple regressions were run with the eye tracking parameters assumed as dependent variables and the BIS-11 total, and subtotal scores as independent variables.

## Results

### Eye Tracking Results

#### Visual Search Strategy (All Subjects)

We investigated the visual search strategy during the reading of financial disclosure documents. The analysis by Entry Time revealed that subjects visually scanned the documents following this sequence: top left – bottom left – top right – bottom right. After the exploration of these main quadrants, individuals revisited some minor AOIs in the following order: *SRRI*, last years of the *Past performance*, more recent years of the *Past performance*, and *Disclaimer.*

**FIGURE 2:**
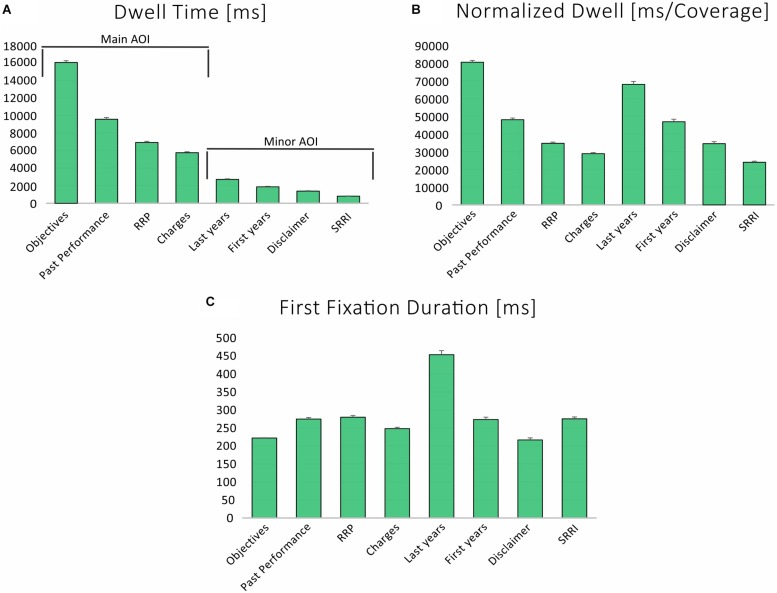
Mean (±SE) values representing **(A)** dwell time (absolute value). **(B)** Dwell time (relative value). **(C)** Normalized dwell (ms/Coverage) for main and minor AOIs, averaged across participants and trials.

The analysis of the time spent to process each AOIs was conducted considering the DT and the DT%, as measures of the absolute and relative time spent for each AOIs, respectively. As revealed by Figure 2A, the section *Objectives* needed the longest time to be processed (DT = 16055 ± 8212 ms; DT% = 39.5% ± 16.1), followed by the section *Past performance* (DT = 9583 ± 7733 ms, DT% = 22.4% ± 14.6), the *RRP* (DT = 6931 ± 5519 ms, DT% = 16.2% ± 11) and the *Charges* (DT = 5764 ± 4622 ms, DT% = 13.6% ± 8.8). When referring to the minor AOIs, subjects spent more time looking at the most recent years (DT = 2723 ± 2706 ms, DT% = 6.8% ± 6.1) than the first years (DT = 1879 ± 2316 ms, DT% = 4.1% ± 4.6) of the *Past performance*, or at the *Disclaimer* (DT = 1388 ± 1893 ms, DT% = 3% ± 3.8) and the *SRRI* (DT = 817 ± 885 ms, DT% = 2.1% ± 2).

The time spent by subjects at exploring the AOIs was computed also taking into account the AOI coverage, i.e., normalizing for AOIs size. As displayed in Figure 2B, the NormDT confirmed that the section *Objectives* needed the longest time to be processed (80536 ms/px% ± 41195), closely followed by the most recent years of the *Past performance* (680275 ± 67592), the initial year of the *Past performance* (46932 ± 57861), the *RRP* (34767 ± 27683), the *Disclaimer* (35549 ± 47213), the *Charges* (28915 ± 23187), and the *SRRI* (24051 ± 26051) (*F* = 489.5, *p* < 0.0001). The highest number of revisits was counted on the *Past performance* (2.8 ± 2.6) and on the *RRP* (2.7 ± 2.1), while the lowest on the SRRI (1.2 ± 1.4) and the *Disclaimer* (1.3 ± 1.7).

Subjects’ AFD across the whole experiment was 300 ms (±219), with the highest value associated to the last years of the *Past performance* (469 ± 333 ms), and the lowest one to the *Disclaimer* (222 ± 206 ms). Congruently, the two above-mentioned AOIs were associated to the highest (453 ± 453 ms) and the lowest (217 ± 300 ms) values of the FFD, respectively, as displayed by Figure 2C.

We did not observe any statistically significant influence of the riskiness of the financial products, as expressed through the SRRI, on the attention distribution toward visual stimuli.

#### Neutral vs. Colored Conditions

**FIGURE 3:**
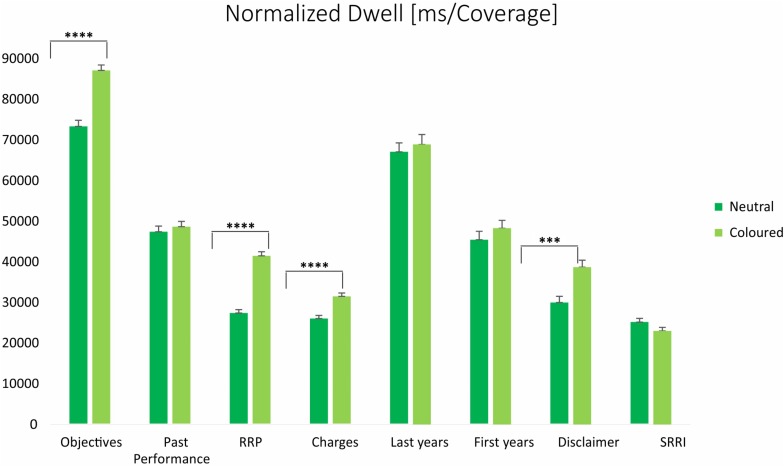
Mean (±SE) values representing the normalized dwell (ms/coverage) for main and minor AOIs, averaged across participants and trials: neutral vs. colored condition. ^*⁣*⁣**^p < 0.0001; ^∗∗∗^*p* < 0.0002.

Individuals’ visual scan strategy, described by Entry time, was not found to be sensitive to colors, since no statistically significant differences were found between the neutral and the colored layouts. On the other hand, the analysis of the processing time revealed that, on average, higher time was spent to observe the colored than neutral stimuli (45136 ± 14641 ms vs. 37965 ± 16884 ms) (*F* = 85.6, *p* < 0.0001). In the colored condition, the DT was higher for the section *RRP* (8273 ± 5868 ms vs. 5466 ± 4694 ms) (*F* = 114.2, *p* < 0.0001), but no statistically significant differences were detected for the *SRRI*, the section *Past Performance* and the subsections of its graph. In addition, a higher DT was observed for the section *Objectives* (17368 ± 7774 ms vs. 14622 ± 8440 ms) (*F* = 47.5, *p* < 0.0001), the *Charges* (6284 ± 4863 ms vs. 5197 ± 4276 ms) (*F* = 23.2, *p* < 0.0001), and the *Disclaimer* (1587 ± 2070 ms vs. 1170 ± 1652 ms) (*F* = 20.4, *p* < 0.0001). These exploratory patterns were confirmed, or even reinforced, when normalizing the Dwell time for the AOI coverage, through the NormD as revealed by Figure 3. We also observed that in the neutral condition, the number of revisits was higher for *Charges* (mean diff: 0.43, *p* = 0.0001), *SRRI* (mean diff: 0.21, *p* = 0.0037), *Past performance* (mean diff: 0.64, *p* < 0.0001), and both the most recent (mean diff: 0.63, *p* = <0.0001) and the last recent years (mean diff: 0.34, *p* = 0.0217) of the time span.

Moreover, the colored condition was associated with higher AFD (326 ± 243 ms vs. 218 ± 185 ms) and FFD (273 ± 289 ms vs. 237 ± 209 ms) values.

**FIGURE 4:**
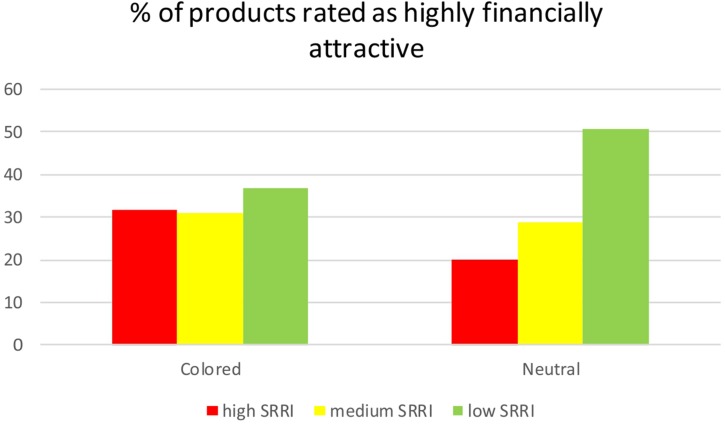
Percentage (%) of products evaluated as highly financially attractive, according to SRRI level (high, medium, and low) and experimental condition (neutral vs. colored).

The analysis of subjects’ evaluation of products financial attractiveness, revealed a significant role of the color. As presented in Figure 4, in the neutral condition, products were evaluated as highly financially attractive in either 20 or 51% cases, respectively, depending on the SRRI high or low; at variance, in the colored conditions, those percentages became 32 and 37%, respectively, with a significantly different distribution of judgment across experimental conditions (*p* = 0.0045).

### Behavioral Results (BIS-11)

The BIS-11 questionnaire was evaluated taking into account standard and inverse scores, and both total and second-order factors. Total BIS-11 average value was 55.5 (±6.7), ranging from a minimum of 39 to a maximum of 70 (Q1 = 51; Q2 = 55; Q3 = 60). The analysis of second-order factors revealed the following average values: 15.2 (±2.5) for *Attentional Impulsivity*, 18.3 (±3.2) for *Motor Impulsivity*, and 22 (±4) for *Non-planning Impulsivity*. No statistically significant between –group differences were found.

### Correlations BIS-11-Eye Tracking

Simple regressions showed that BIS-11 total score was inversely related to the AFD (*p* < 0.0001) and FFD (*p* = 0.0002), in the neutral condition, though not in the colored condition. A positive relationship was also found between the number of revisits and the BIS-11 (*p* < 0.0001), which was significant for both the colored, and the neutral conditions (*p* = 0.0004 and *p* < 0.0001, respectively). The multivariate analysis, where NormD, FFD, and AFD were alternatively included as dependent variables, confirmed the independent role of the experimental condition, the AOI type and the BIS-11 total score in modulating attention allocation. Table 1 displays the results of such analysis with reference to the AFD. No significant associations were found between BIS-11 second-order factors and eye tracking parameters, in the regression analysis.

**TABLE 1:** Multivariate analysis results with AFD as dependent variable. Experimental condition, AOI type, and BIS-11 score were taken as independent ones.

	**Coef**	**Std. Error**	**Coef/SE**	**Chi-Square**	***P*-Value**	**Exp (coef)**	**95% Lower**	**95% Upper**
Constant	6.356	0.433	14.665	215.077	<0.0001	575.845	264.246	1, 346.609
BIS-11	–0.066	0.007	–9.19	84.455	<0.0001	0.936	0.923	0.95
AOI *objectives*	0.505	0.129	3.901	15.219	<0.0001	1.657	1.286	2.135
AOI *past performances*	0.721	0.137	5.28	27.876	<0.0001	2.057	1.574	2.688
AOI *charges*	0.608	0.133	4.584	21.012	<0.0001	1.837	1.416	2.383
Neutral condition	–1.045	0.111	–9.448	89.26	<0.0001	0.352	0.283	0.437

*AFD, average fixation duration; AOI, area of interest.*

## Discussion

The main aim of the present research was to investigate individuals’ attention allocation during the processing of financial disclosure documents drafted, in an ecological manner, in the pre-determined format imposed by the UCITS IV Directive and displaying real financial data. The use of eye tracking for the investigation of financial information processing represents the original aspect of this research. In a previous study, Ceravolo et al. (2018) analyzed oculomotor behavior of non-professional investors observing that attention distribution across the different sections of the document is strongly influenced by the presentational layout. Authors highlighted that the format displaying the section *Costs* as first piece of information, in the top-left quarter increases the time spent to process the whole document.

In this study, we sought to test the hypothesis that stimulus basic components such as the color could affect individuals’ attention even in case of financial information processing. The study of the influence of colors on individuals’ central and autonomic nervous system has a long tradition (Goldstein, 1942; Buechner and Maier, 2016), and, recently, several researchers have started to adopt an interdisciplinary vision to apply the findings from neuroscience to other fields such as architecture, interior design, marketing, communication. By way of examples, Küller et al. (2009) have studied the effect of colored room interiors on physiological state, mood, and performance. Surprisingly, to the best of our knowledge, no study has investigated this aspect in the financial decision-making, exploiting a neuroscientific approach. With this research, we aimed at filling this gap, through reproducing the official financial disclosure document.

Our analysis on the influence of colors on individuals’ visual exploratory strategy confirms the hypothesis tested: all eye-tracking parameters concur in highlighting that the KIIDs with colored sections are observed for longer time than black and white KIIDs. This finding is independent from both individuals’ traits as impulsivity and demographic features. The observed influence of color is especially interesting, as the KIID is not expected to elicit any emotional reaction, not being marketing material, in agreement with the Directive. In fact, in a traditional economic perspective, which hypothesizes “rational” decision makers, it is assumed that no affective reactions at all should arise while being exposed to financial information. Conversely, we observe that the time dedicated by subjects to process the whole document is extended by the addition of color to a few data sections. In such cases, individuals’ attention is higher not only toward the colored sections, but also toward the ones still draft in black and white, such as the *Objectives* and the *Charges*.

Interestingly, the influence of color is not limited to the attention allocation process, but it also concerns the financial choice. In fact, the analysis of the relationship between the SRRI level, the perceived attractiveness of financial products and the experimental condition, revealed that the colored KIIDs seem to reduce the individual anchoring to the SRRI, during financial information processing. Indeed, while in the neutral condition, subjects’ evaluations display an inverse relationship with the SRRI level, with a higher portion of products rated as highly financially attractive when the SRRI is low, compared to when the SRRI is high or medium, in the colored condition, the same proportion of products are perceived as highly financially attractive independent from the SRRI. This result might suggest that the higher attention triggered by the color also translates in more conservative decisions, alerting the subjects’ who reduces the dependence of his judgment from the synthetic SRRI index, while processing all the other sources of information. These findings are important in two different ways. Firstly, they highlight that a change in a graphic aspect of a piece of information can affect not only the attention allocation process but also financial decision-making outcome clearly pointing out to the need to incorporate the influence of contextual factors, largely disregarded in the financial theories until now. Secondly, the observation that color triggers higher level of attention but is associated to a lower sensitivity to the risk/reward profile of the financial product, smoothing choices, suggests the urgency to start to investigate the specificities of the link between attention and decision outcomes, also in finance, since the assumption of a linear relationship might be an oversimplification.

An extensive literature outlines the existence of a relationship between attention and impulsivity, that has been found to influence performance on a wide array of cognitive tasks (Anderson and Revelle, 1983, Dickman, 1985, Brunas-Wagstaff et al., 1994). Attention disorders are supposed to originate from an impaired subject’s ability to inhibit behaviors, feelings or thoughts, and that distract them from goal achievement (Nigg, 2001). In our research, we sought to consider the influence of the impulsivity personality trait on oculomotor behavior, while subjects examined financial documents. Our analysis helps to predict that the more impulsive the subject the shorter the time he takes to scan the document. This association evokes what is expected based on studies conducted on many neuropsychiatric conditions, as attention-deficit/hyperactivity disorder (ADHD), autism spectrum disorder, or intellectual disability. In such studies, specific patterns of attentional mechanism impairment, by age and disease, have been outlined (Cortese et al., 2012). In our sample of healthy young subjects, we found that BIS-11 total scores were inversely related to the first and average fixation duration. Interestingly, such association was highly significant when subjects were exposed to black and white KIIDs, though disappeared in presence of colored documents. This evidence suggests that the increased attention induced by color is strong enough to compensate for individual impulsivity. This aspect is especially relevant for regulators, suggesting that basic stimulus features – even in the financial domain – can be modulated in order to enhance individual attention toward important documents, helping to deal with the heterogeneity of readers’ personality. Previous studies have shown the existence of a positive correlation not only between impulsiveness and economic and financial decisions (as when learning is required, e.g., in the Iowa Gambling Task) (Franken et al., 2008; Sweitzer et al., 2008), but also with respect to other risky behaviors, like drug abuse, aggression, drunk driving, and unprotected sex (Stanford et al., 1996). This evidence could provide the basis for analyzing the relationship between impulsivity and the processing of any type of information disclosure material. For instance, tobacco package warning messages and pictures illustrating the negative health effects could be presented in more vivid colors. Moreover, beside healthy population, impaired impulsivity is a critical factor associated to dysfunctional situations as pathological gambling and research and clinical expertise agree in considering impulsivity as integral to its full understanding (Alessi and Petry, 2003) since it has been found to predict gambling frequency (Benson et al., 2012) and increase the risk of gambling onset in youths with low socio-economic status (Auger et al., 2010). The existence of a link between impulsivity and gambling calls attention to the urgency to develop innovative intervention strategies directed at managing the impulsivity personality trait. In showing how the introduction of a simple contextual factor as the color is able to modulate attentional mechanisms compensating the influence of impulsivity, our study should therefore be of interest for policy-makers and financial regulators. Surely, several other individual variables can also modulate attention allocation: however, our sample was reasonably homogeneous with respect to characteristics like age, educational background, professional experience, or data analysis skills, and all these variables resulted to be evenly distributed in the groups exposed to either neutral or colored KIIDs.

As a possible consequence of subgroup similarity, the subjects’ exploratory behavior was similar in both the neutral and colored condition and in line with what was already described by Ceravolo et al. (2018) in a similar experience. In detail, the scan path always proceeded from top left quarter to bottom left and then from top right to bottom right, as usually happens during the reading of any written document, in European languages. Moreover, attention distribution was significantly different across the four main AOIs, in spite of their equal size, in both experimental conditions: longer time was dedicated to the sections *Objectives* and *Past performance*, than to *Charges* and *RRP*. In particular, a higher average fixation duration was detected for these two AOIs, suggesting that a greater cognitive effort was required to subjects to scan them. Indeed, the section *Objectives* presents a narrative description of financial information, while the section *Charges* displays data in a tabular form (hence easier to explore and summarize); moreover, the section *RRP* synthetizes the information through the graphical risk scale, which might help in developing a heuristic to simplify the reading process. Conversely, even if the section *Past performance* is a graphical representation, its 10 years comparison, year by year, to a benchmark, does not permit an immediate understanding, thus requiring a longer processing. Interestingly, the SRRI, one of the sub-sections of the document, which could be considered the interpretation key of the whole document, does not grab a great amount of subjects’ attention.

The greater attention dedicated to the minor AOI represented by the last years of the *Past performance* is in line with a financial perspective which considers them important as they refer to a crucial time period: in fact, the product’s trend across the most recent years usually affect investors’ decision making through inducing a *recency (or end-anchoring)* bias. Our results corroborate previous findings in neurofinance which demonstrate an attentional bias toward the last portion of a graph which displays financial assets past performances (Hüsser and Wirth, 2014; Duclos, 2015).

Surprisingly, *SRRI* appears a less relevant input for non-professional investors, even after normalizing for the AOI size. There are different possible interpretations of this finding. The first is that a graphic representation of the risk and reward associated with the financial products on a 7-point scale completely fulfils its role of conveying the information in an instantaneous way; therefore, a very short time is needed to grab it. A second interpretation suggests that the *Past performance* is not predictive of futures ones, as it is specified in the *Disclaimer*, so that the SRRI is merely considered a rapid information about the volatility level of past returns obtained by the fund management. Therefore, the very short fixation time needed to process this AOI could disclose the individuals’ low interest toward such piece of information. The first interpretation would have been supported by the detection of high fixation duration values (either FFD or AFD) associated to the SRRI. The fact that these two proxies of individuals’ attentional effort are close to the minimum values rather than to the maximum ones, seems to reinforce the second interpretation, suggesting that subjects tend to infer the attractiveness of the products from other sources of information, such as the fund’ returns during the most recent years. On the other hand, the inverse relationship detected in the neutral condition between the level of SRRI and evaluation of products’ financial attractiveness seems to point out to a relevant role of that piece of information in modulating individuals’ behavior. A further development of this study should try to clarify the influence exerted by summary indicators of the risk-reward profile of financial products on the individual’s attention allocation process and, in turn, on his/her final judgment about products’ attractiveness.

Visual attention is a scarce resource, cognitive effortful and selective: a continuous automatic competition occurs among visual stimuli, to speed up the representation of the observed object (or text, picture) in the visual cortex. Since previous research on visual attention demonstrated that pictorial information grabs greater attention than text (Rayner et al., 2001), some researchers, like Hüsser and Wirth (2014), explored the effects of converting the narrative description of investment philosophy into a graphic representation. They quantified the attention dedicated to the investment philosophy comparing it to the time taken by the same subjects to explore the graphical representation of the fund past performance, observing that the former attracted less attention than the latter. In their experiment, however, subjects were alternatively exposed to these two different information sources, that therefore did not compete to grab subjects’ attention. We sought to solve this limitation arguing that in order to investigate visual search behavior in a real-world scenario, it would have been more methodologically sound to present complex stimuli displaying all sources of information at once. In this way, the competition between different information types would emerge and allow speculations about attention modulation by stimulus features.

Overall, these findings underpin the need to study individual financial behavior considering the influence of automatic processes and emotions on the initial phase of information processing and in the subsequent ones of the decision making process. Our results are of primary importance for three main actors of financial markets. Firstly, results could inform regulators involved in the design of disclosure documents that aim to raise investors’ protection. Secondly, our evidence could provide support to subjects involved in developing financial products marketing strategies. Finally, they could help investors at increasing their awareness about the influence of basic features of the documents on their attention.

This study has also some limitations. The most critical one is that subjects were not involved in real financial choices. The artificial condition we created to conduct this experimental research does not represent a real-life situation, with all the problems of external validity typical of experimental economics and finance. Previous studies have highlighted the importance to study the decision-making process in a real environment, with subjects using their own financial resources, suggesting the importance and the feasibility of re-creating conditions very close to the reality (Raggetti et al., 2017). Although the protocol did not mimic a real world scenario, we tried to link the attention allocation process to a decision outcome, by asking participants to express a first judgment on the financial attractiveness of the products. In a following study, it could be interesting to explore whether visual search strategy and real financial choices are different from those we observed and how they are modulated by the level of income and of financial education. Moreover, our investigation into the attention mechanisms and their relationship with financial information processing did not include the assessment of neurophysiological proxies of arousal, as the skin conductance response and pupil dilation. Extracting and analyzing pupil size time course during the KIID visual scan would have likely provided useful information about attention (e.g., Vossel et al., 2015; Lasaponara et al., 2019) and cognitive effort (e.g., van der Wel and van Steenbergen, 2018). We acknowledge that further developments of the present research should take into account such measures.

Future research in neurofinance could also investigate whether individuals’ sensitivity to colors (proxied through eye tracking metrics recorded while subjects observe colored stimuli which do not contain any information) affects the patterns of attention allocation we reported. It would also be interesting to study the influence of complementary colors (i.e., the opposite hues on the color wheel) on oculomotor patterns applied to the same document. Finally, further developments of this study could integrate the eye tracking analysis with other investigation techniques, such as the detection of skin conductance response, aiming at clarifying whether the higher attention dedicated to colored information is triggered by an increased arousal.

## Ethics Statement

This study was carried out in accordance with the recommendations of the Institutional Review Board of the University of Rome Tor Vergata with written informed consent from all subjects. All subjects gave written informed consent in accordance with the Declaration of Helsinki. The protocol was approved by the Institutional Review Board of the University of Rome Tor Vergata.

## Author Contributions

All authors conceived the experimental design, wrote the manuscript, and approved the final manuscript. VF, LL, and LF carried out the experiment and collected the data. RC analyzed the data on the impulsivity personality trait. LF and MC analyzed and interpreted the eye tracking data. GR critically revised the manuscript.

## Conflict of Interest Statement

The authors declare that the research was conducted in the absence of any commercial or financial relationships that could be construed as a potential conflict of interest.
